# Research on Signal Denoising of Pumped-Storage Units Based on Parameter-Adaptive VMD and Wavelet Thresholding

**DOI:** 10.3390/s26133974

**Published:** 2026-06-23

**Authors:** Tianmin Li, Yuechao Wu, Fengque Pei

**Affiliations:** 1 College of Mechanical and Electrical Engineering, Hohai University, Changzhou 213200, China; 2 Powerchina Huadong Engineering Corporation (China), Hangzhou 311122, China

**Keywords:** pumped-storage units, signal denoising, VMD, IPSO, comprehensive fitness function

## Abstract

To address the non-stationary and non-linear characteristics of vibration signals collected by sensors in pumped-storage units, as well as their susceptibility to strong background noise interference, this paper proposes a joint signal denoising method combining parameter-adaptive Variational Mode Decomposition (VMD) and wavelet thresholding. First, the Improved Particle Swarm Optimization (IPSO) algorithm is utilized to adaptively optimize the key parameters of VMD using a comprehensive fitness function as the objective, thereby achieving the optimal decomposition of the signal. Subsequently, a cross-correlation analysis method is introduced to screen the decomposed components, followed by a secondary denoising process using a wavelet threshold to accomplish the final signal denoising. Experimental validations using simulated run-out signals and field-measured sensor data from a pumped-storage power station, along with comparisons against other methods, demonstrate that the proposed method can eliminate noise more effectively. It significantly improves the signal-to-noise ratio (SNR) and reduces the root mean square error (RMSE). Consequently, this study provides a reliable data foundation for the subsequent research and analysis of the units, demonstrating substantial practical engineering significance.

## 1. Introduction

With the advancement of the global “dual-carbon” goal, the core position of hydropower as a clean and renewable energy source has become increasingly prominent. Pumped-storage power stations undertake key tasks such as peak shaving and valley filling, frequency regulation, and phase regulation, and constitute an important component in constructing a new-type power system [1,2]. As the core project of China’s “West-to-East Power Transmission” and the world’s largest diversion-type hydropower station, the Yarlung Zangbo River Hydropower Station is progressively advancing its construction. It has planned a 12-million-kilowatt large-scale pumped-storage power station as a supporting facility to enhance power system stability, aiding in the construction of the clean energy base in Southwest China and the implementation of the “dual-carbon” target. The pump turbine serves as the core equipment of the pumped-storage power station supporting the Yarlung Zangbo River Hydropower Station. Its operational state directly relates to the safe and stable operation of the entire Yarlung Zangbo River hydropower base and the reliability of power transmission. Due to the complex geological conditions and special hydrological environment of the Yarlung Zangbo River basin, the supporting pumped-storage units must switch frequently between pump operation mode and turbine operation mode. During operation, the units are inevitably subjected to coupled excitations from multiple factors, including hydraulic, mechanical, and electromagnetic forces, thereby producing complex vibration signals. These vibration signals, acquired by vibration sensors, contain abundant information regarding the equipment’s health status, serving as a crucial basis for condition monitoring and fault diagnosis.

However, compared to conventional hydropower units, the raw vibration signals collected by sensors of pumped-storage units in actual situations often exhibit stronger non-linear and non-stationary characteristics [3,4], and are contaminated by intense background noise. This noise often submerges the fault characteristic frequencies, resulting in an extremely low signal-to-noise ratio, which severely affects the accuracy of subsequent feature extraction and the reliability of fault diagnosis. Therefore, performing signal denoising processing on them to effectively separate and extract components containing fault features from a strong noise background is of vital practical significance and engineering value for subsequent unit signal feature extraction, fault diagnosis, and ensuring the safe and stable operation of major national hydropower projects.

A comprehensive review of a large amount of relevant literature reveals that, currently, signal analysis methods targeting such rotating machinery mainly include the following: Short-Time Fourier Transform (STFT), wavelet transform (WT) [5,6], empirical mode decomposition (EMD) [7], ensemble empirical mode decomposition (EEMD) [8,9], and Variational Mode Decomposition (VMD) [10]. The EMD and its improved algorithm EEMD proposed by Huang et al. [11,12] can adaptively decompose signals and are widely used in rotating machinery fault diagnosis. Wang et al. [13] separated draft tube pressure signals using the ensemble empirical mode decomposition method, and combined it with autocorrelation analysis and threshold denoising to denoise the signals. Yin et al. [14] utilized an improved EEMD to isolate intrinsic noise hidden in original signals. Pan Tianhang et al. [15] proposed an improved EMD-based fault feature extraction method for vibration signals of hydropower units, and used this algorithm to analyze the run-out signals of abnormal vibration in a hydropower station, which could effectively shield interference signals. However, studies by North American scholars have further corroborated that EMD and EEMD possess inherent defects such as endpoint effects and mode mixing, making it difficult to achieve precise signal separation. Even with algorithmic improvements, such issues cannot be completely eradicated [16]. To compensate for the shortcomings of a single EMD-based algorithm, overseas researchers proposed a multi-layer denoising approach combining EEMD and wavelet thresholding, which significantly improves signal processing accuracy through mode screening and secondary denoising, achieving favorable results [17].

In 2013, Dragomiretskiy proposed a non-recursive signal decomposition method—Variational Mode Decomposition (VMD). This method boasts high decomposition efficiency and is highly applicable in non-linear signal research [18]. Compared to EMD, VMD can effectively suppress mode mixing. Tian Weikun et al. [19] proposed a vibration signal denoising method for hydropower units combining VMD and an improved wavelet threshold, and verified through experimental analysis that the proposed method effectively reduces the root mean square error of signals and significantly enhances the denoising effect. Zheng Xianghao et al. [20] proposed a process suitable for shaft vibration signal denoising based on wavelet threshold denoising, VMD, and cross-correlation analysis. Despite the significant advantages of VMD, its decomposition performance heavily depends on the pre-setting of two key parameters: the number of modal decompositions *K* and the quadratic penalty factor α. Traditional methods usually determine parameters based on experience or trial and error, making it difficult to ensure optimal decomposition. If parameters are chosen improperly, mode mixing may also occur, preventing effective signals from being accurately separated from noise, and affecting subsequent research. Bagri et al. [21] specifically investigated the drawbacks of manual parameter tuning in VMD, pointing out that subjective trial-and-error parameter selection is a core pain point restricting the engineering application of VMD, and parameter-adaptive optimization schemes are urgently needed.

In recent years, a vast number of scholars have dedicated themselves to utilizing optimization algorithms (such as Particle Swarm Optimization (PSO), whale optimization algorithm (WOA), etc.) with indicators like envelope entropy, permutation entropy, or kurtosis as the fitness function to adaptively optimize VMD parameters, achieving notable results. The theoretical framework proposed by Antoni [22] based on information entropy (Infogram) to capture repetitive transient impact features profoundly reveals the superiority of entropy indicators in measuring the clarity of periodic impulses under strong background noise, thereby providing solid mathematical and physical support for the parameter-adaptive optimization of decomposition algorithms such as VMD. Huang et al. [23] proposed a method to optimize VMD parameters based on scale-space. Lin [24] proposed optimizing VMD parameters based on CS. Wei Ankai [25] proposed an optimized SSA-VMD denoising method for the issue of rolling bearing fault signal denoising, verifying its effectiveness through simulations and actual bearing fault signals respectively. Wang et al. [26] utilized the whale optimization algorithm to adaptively determine the parameters in VMD decomposition, successfully extracting the fault features of rolling bearings. Wang Weiyu et al. [27] also adopted the GSA-optimized VMD method to decompose and reconstruct signals during the fault diagnosis of hydropower units, successfully extracting feature information. Although the aforementioned methods can obtain relatively reasonable parameter combinations for VMD decomposition, inappropriate selection of the fitness function sometimes causes the optimization algorithm to easily fall into local optimal solutions, and a small amount of high-frequency noise may still remain in the effective modal components obtained by VMD decomposition. Therefore, hybrid denoising strategies combining VMD with other signal processing techniques have become a research hotspot.

In summary, although VMD-based denoising methods have made certain progress, how to construct a more precise objective function for VMD parameter optimization—to effectively separate and extract the primary frequencies from the vibration signals of pumped-storage units and achieve superior denoising—remains to be further explored. To address this gap, this paper proposes a joint signal denoising method combining parameter-adaptive VMD and wavelet thresholding. Specifically, the proposed method first employs an Improved Particle Swarm Optimization (IPSO) algorithm to optimize the VMD parameters, enabling adaptive signal decomposition. Subsequently, wavelet thresholding is introduced to perform secondary denoising on the screened sensitive modes to further enhance the signal-to-noise ratio (SNR). Finally, the processed components are reconstructed to restore the authentic signal characteristics of the unit. Experimental validations are conducted using both simulated pump turbine run-out signals and field-measured water guide bearing run-out data. Comparisons with other denoising methods further verify the effectiveness of the proposed approach, thereby providing novel insights for the research and analysis of signal denoising in pumped-storage power stations.

## 2. Introduction to Basic Principles

### 2.1. VMD Principle

VMD is an adaptive signal processing method capable of decomposing a signal into multiple Intrinsic Mode Function (IMF) components corresponding to different central frequencies. The main process is as follows [28].

First, the Hilbert transform is used to calculate the unilateral spectrum of each modal component to obtain its analytic signal. Then, an exponential term is introduced to mix the analytic signals of each mode, modulating their spectra to the baseband, and the squared norm of the modulated signal is utilized to estimate the bandwidth of each mode. The constrained variational model is as follows:

(1) $$\min_{\left\{u_{k}\right\},\left\{\omega_{k}\right\}}\left\{\sum_{k}{∥\partial_{t}\left[\left(\delta \left(t\right)+\frac{j}{\pi t}\right)*u_{k}\left(t\right)\right]e^{-j\omega_{k}t}∥}_{2}^{2}\right\}$$

where $\left\{u_{k}\right\}$ represents the set of all modal components; $\left\{\omega_{k}\right\}$ represents the central frequencies corresponding to each mode; and δ(t) is the Dirac function.

A quadratic penalty factor α and a Lagrange multiplier λ are introduced to transform the constrained problem into an unconstrained one. The augmented Lagrangian function is

(2) $$\begin{array}{cc} L(\left\{u_{k}\right\},\left\{\omega_{k}\right\},\lambda )= & \;\alpha \sum_{k}{∥\partial_{t}\left[\left(\delta \left(t\right)+\frac{j}{\pi t}\right)*u_{k}\left(t\right)\right]e^{-j\omega_{k}t}∥}_{2}^{2}\\ \; & +{∥f\left(t\right)-\sum_{k}u_{k}\left(t\right)∥}_{2}^{2}+\langle \lambda \left(t\right),f\left(t\right)-\sum_{k}u_{k}\left(t\right)\rangle \end{array}$$

The Alternating Direction Method of Multipliers (ADMM) is utilized to iteratively update the modal components, central frequencies, and multipliers for Equation (2) until the iterative convergence condition in Equation (3) is satisfied, at which point the iteration stops, and *K* components are ultimately obtained:

(3) $$\sum_{k}\frac{∥{\hat{u}}_{k}^{n+1}-{\hat{u}}_{k}^{n}{∥}_{2}^{2}}{∥{\hat{u}}_{k}^{n}{∥}_{2}^{2}}<\epsilon$$

where ϵ represents the solution accuracy. The updates for ${\hat{u}}_{k}$ and $\omega_{k}$ are respectively

(4) $${\hat{u}}_{k}^{n+1}\left(\omega \right)=\frac{\hat{f}\left(\omega \right)-\sum_{i\neq k}{\hat{u}}_{i}\left(\omega \right)+\frac{\hat{\lambda }\left(\omega \right)}{2}}{1+2\alpha {\left(\omega -\omega_{k}\right)}^{2}}$$

(5) $$\omega_{k}^{n+1}=\frac{\int_{0}^{\infty }\omega {\left|{\hat{u}}_{k}^{n+1}\left(\omega \right)\right|}^{2}d\omega }{\int_{0}^{\infty }{\left|{\hat{u}}_{k}^{n+1}\left(\omega \right)\right|}^{2}d\omega }$$

### 2.2. Wavelet Threshold Denoising

Wavelet threshold denoising is based on the multi-resolution characteristics of the wavelet transform and the sparsity of signals. The primary steps of its algorithm are as follows.

Step 1 Wavelet decomposition. Select an appropriate wavelet basis function and decomposition level, perform wavelet decomposition on the signal, and obtain the approximation coefficients and detail coefficients for each level.

Step 2 Threshold quantization processing. For the high-frequency detail coefficients of each level, calculate the threshold, and use the selected threshold function to process the coefficients to obtain the estimated wavelet coefficients.

Step 3 Wavelet reconstruction. Perform the inverse discrete wavelet transform utilizing the processed wavelet coefficients and the original low-frequency coefficients to reconstruct the denoised signal.

The soft threshold function is employed in the wavelet denoising algorithm in this study. It possesses good continuity, and the reconstructed signal is relatively smooth. The threshold is determined using the universal threshold calculation formula.

### 2.3. Improved Particle Swarm Optimization (IPSO)

The PSO algorithm simulates the foraging behavior of bird flocks. The essence of its optimization problem is to determine the position of the optimal solution in space through the movement direction and velocity of particles [29]. Assuming that in a *D*-dimensional search space, the particle population size is *N*. The position vector of the *i*-th particle is $X_{i}$, and the velocity vector is $V_{i}$. In each iteration, the optimal position experienced by the individual is denoted as $P_{i}$, and the global optimal position of the entire particle swarm is denoted as $P_{g}$. The velocity and position of each particle are updated according to the following equations:

(6) $$\begin{array}{cc} V_{id}^{t+1}= & \;wV_{id}^{t}+c_{1}r_{1}\left(P_{id}^{t}-X_{id}^{t}\right)+c_{2}r_{2}\left(P_{gd}^{t}-X_{id}^{t}\right) \end{array}$$

(7) $$X_{id}^{t+1}=X_{id}^{t}+V_{id}^{t+1}$$

where *t* is the number of particle swarm iterations; $r_{1}$ and $r_{2}$ are mutually independent random numbers between 0 and 1; $V_{i}$ and $X_{i}$ are the velocity and position of the *i*-th particle, respectively; $P_{i}$ and $P_{g}$ are the individual and global optimal positions, respectively; *w* is the inertia weight; and $c_{1}$ and $c_{2}$ are learning factors.

To better balance the algorithm’s global and local search capabilities, this paper adopts the Improved Particle Swarm Optimization algorithm, which utilizes a non-linear dynamic inertia weight coefficient. The formula is as follows:

(8) $$w=w_{min}+\left(w_{max}-w_{min}\right)\cdot e^{-Dec\cdot {\left(t/T_{max}\right)}^{2}}$$

where $w_{max}$ and $w_{min}$ denote the maximum and minimum values of the inertia weight; $T_{max}$ represents the maximum number of iterations; and Dec is the decay factor, a positive number less than 1.

Compared to PSO, IPSO realizes the adaptive adjustment of the inertia weight. In the initial stage, *w* is large and decreases slowly, ensuring sufficient global search; in the later stage, it decreases rapidly, enhancing local convergence performance.

### 2.4. IPSO-VMD Parameter Optimization

The key to utilizing the IPSO algorithm for VMD signal decomposition lies in finding two optimal parameters: the number of modal decompositions *K* and the penalty factor α. Both directly affect the decomposition effect of VMD. If the *K* value is too large, it leads to over-decomposition phenomena; if the *K* value is too small, effective signal separation may be incomplete, mixing multiple modes together and causing under-decomposition. If the penalty factor value is too large, it causes the modes to be too sparse, and if it is too small, it leads to an excessively wide bandwidth [30].

During the VMD parameter optimization process in this paper, in order to avoid over-decomposition and under-decomposition phenomena, and simultaneously to avoid the particle swarm falling into a local optimal solution during the optimization process as much as possible, this research constructs a comprehensive fitness function integrating the Envelope Entropy function, Orthogonality analysis, and Residual Energy as the optimization indicator for the IPSO algorithm. Compared to using only Minimum Envelope Entropy as the fitness function, employing a comprehensive fitness function of all three can more effectively and accurately optimize the algorithm to obtain the best decomposition parameters.

Envelope Entropy: Envelope Entropy (EE) is an important indicator for measuring the sparsity and regularity of a signal. The smaller the entropy value, the more concentrated the energy distribution in the signal envelope spectrum, and the less the noise interference. The calculation formula is

(9) $$EE=-\sum_{i=1}^{N}p_{i}\ln p_{i}$$

where $p_{i}$ is the normalized probability distribution.

Orthogonality Index: The Index of Orthogonality (IO) is used to measure the degree of mutual independence between modal components after decomposition. The closer the IO value is to 0, the better the orthogonality of each component in the time domain, and the lower the degree of mode mixing. Its calculation formula is as follows:

(10) $$IO=\sum_{i=1}^{K}\sum_{j=1,j\neq i}^{K}\frac{\left|\langle u_{i}\left(t\right),u_{j}\left(t\right)\rangle \right|}{∥u_{i}{\left(t\right)∥}_{2}{∥u_{j}\left(t\right)∥}_{2}}$$

where 〈·,·〉 represents the inner product, and ${∥\cdot ∥}_{2}$ represents the $L_{2}$ norm.

Residual Energy Ratio: The Residual Energy Ratio (RER) measures the completeness of the decomposition by calculating the energy proportion of the difference between the original signal and the reconstructed signal. The smaller its value, the closer the decomposed signal is to the original signal, with less loss, retaining the vast majority of the energy of the original signal. The calculation formula for the Residual Energy Ratio RER is as follows:

(11) $$RER=\frac{{∥f\left(t\right)-\sum_{k=1}^{K}u_{k}\left(t\right)∥}_{2}^{2}}{{∥f\left(t\right)∥}_{2}^{2}}$$

In order to achieve the ideal effect of VMD decomposition, for envelope entropy, the focus is mainly on feature clarity, and the smaller the value, the better; for the orthogonality index, the smaller the value, the better, indicating a low degree of mode mixing; and for the energy residual ratio, its value is also better the smaller it is, indicating that the signal decomposition is sufficient, mode loss is avoided, and signal integrity is guaranteed. To achieve multi-objective collaborative optimization, a comprehensive fitness function is constructed based on the above three indicators as follows:

(12) $$Fitness=\gamma_{1}\cdot \bar{EE}+\gamma_{2}\cdot IO+\gamma_{3}\cdot RER$$

where $\bar{EE}$ is the average envelope entropy, and $\gamma_{1},\gamma_{2},\gamma_{3}$ represent weighting coefficients.

Here, the comprehensive fitness function adopts the linear weighted summation method, following three core principles: the signal magnitude alignment principle, the research objective priority principle, and the engineering experience principle. The numerical magnitudes of the three evaluation indicators vary greatly; therefore, weighting coefficients are required to pull the three indicators to the same optimization magnitude, allowing each indicator to make an effective contribution to the fitness function. Based on the engineering requirements of VMD signal decomposition and denoising, there are clear priorities for the optimization objectives: the primary objective is to suppress mode mixing (dominated by IO); secondly, over-decomposition or under-decomposition must not occur in order to eliminate mixing, and core vibration characteristics such as the rotational frequency and harmonic frequencies of the unit must be preserved to avoid mode loss and ensure signal integrity (dominated by RER); finally, based on the above constraints, the feature clarity is further enhanced (dominated by EE). The weights are selected according to this priority: $\gamma_{2}>\gamma_{3}>\gamma_{1}$. According to the principle of domain engineering experience, the general reasonable value ranges of the three weights are delineated, and then within this range, combined with the specific characteristics of the pump-turbine vibration signal, multiple sets of comparative tests and parameter fine-tuning are conducted to ultimately determine the appropriate weighting coefficients.

**Figure 1:**
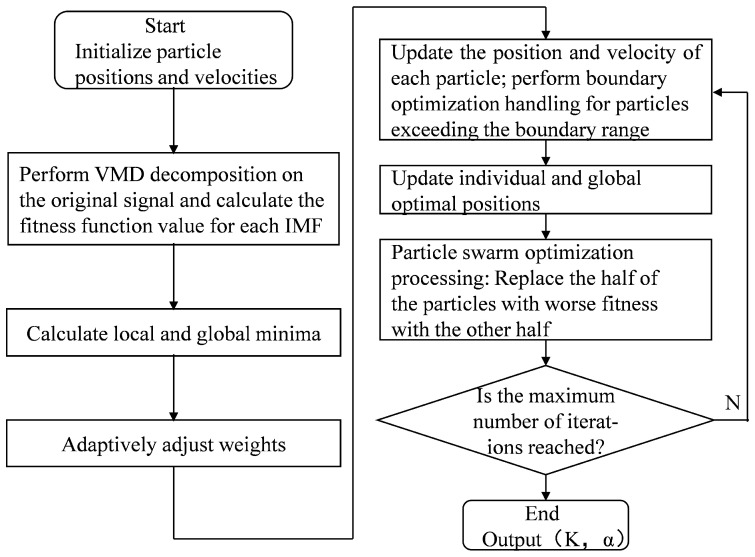
IPSO−VMD optimization flowchart.

Therefore, in summary, the process of VMD parameter optimization is transformed into a problem of finding the best comprehensive fitness function value using IPSO. The IPSO-VMD optimization process is shown in Figure 1.

### 2.5. IPSO-VMD-Wavelet Threshold Joint Denoising

This paper proposes a denoising method based on Improved Particle Swarm Optimization for VMD parameters combined with Wavelet Thresholding. First, the IPSO algorithm is used to perform VMD decomposition on the signal to obtain the optimal two parameters, achieving the purpose of accurately separating target frequencies. Then, utilizing the cross-correlation analysis method, the cross-correlation coefficient between each mode and the original signal is calculated (the closer the absolute value is to 1, the greater the degree of correlation with the original signal).

Aiming at the characteristics of the run-out signals of pumped-storage units, generally speaking, the IMF components dominated by the main characteristic frequencies are located in the low-frequency band, and their correlation coefficients with the original signal are relatively large; the noise-dominated IMF components are usually located in the high-frequency band, and their correlation coefficients with the original signal are relatively small. Therefore, a coefficient threshold can be set to separate effective modal components and noise-dominated components. After removing the noise components, secondary denoising is performed on the effective components. Effective components with large correlation coefficients are retained directly, while those with smaller correlation coefficients undergo wavelet threshold processing. Finally, all denoised effective modal components are reconstructed to obtain the ultimate denoised signal.

In this process, the calculation formula for the cross-correlation coefficient is as follows:

(13) $$\rho =\frac{\sum_{i=1}^{N}\left(x_{i}-\bar{x}\right)\left(y_{i}-\bar{y}\right)}{\sqrt{\sum_{i=1}^{N}{\left(x_{i}-\bar{x}\right)}^{2}\sum_{i=1}^{N}{\left(y_{i}-\bar{y}\right)}^{2}}}$$

where $x_{i}$ and $y_{i}$ are the amplitudes of the original signal and the *i*-th modal component at the *N*-th sampling point respectively; and *N* is the number of sampling points.

**Figure 2:**
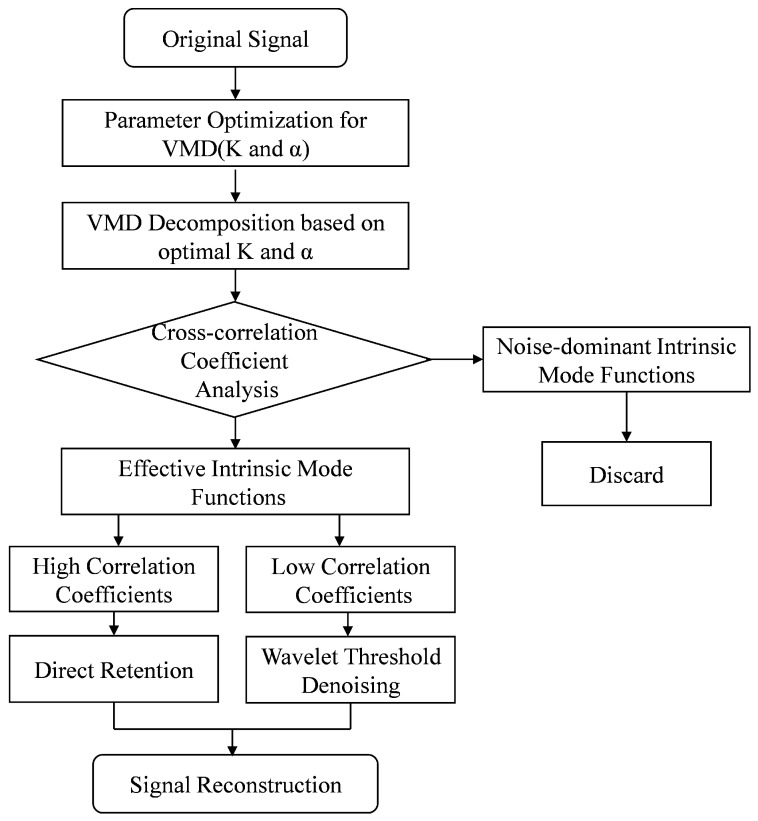
Denoising flowchart of this paper.

Based on the above contents, the flowchart of the denoising method in this paper is illustrated as shown in Figure 2.

## 3. Simulation Run-Out Signal Denoising Analysis

### 3.1. Construction of Simulated Run-Out Signal

In the simulated run-out signals of pump turbine in pumped-storage units, mechanical and hydraulic factors play a dominant role. The frequency of mechanical vibration signals is generally dominated by 1×, 2×, and 3× the unit rotational frequency, while the frequency of hydraulic excitation signals is generally dominated by low-frequency signals. Based on the characteristics of the pump turbine run-out signal, the expression constructed in this paper is as follows:

(14) $$\begin{array}{cc} S(t)= & 0.25\sin\left(2\pi \cdot 0.45f_{n}t\right)+\sin\left(2\pi f_{n}t\right)\\ \; & +0.25\sin\left(2\pi \cdot 2f_{n}t\right)+0.1\sin\left(2\pi \cdot 3f_{n}t\right)+n\left(t\right) \end{array}$$

**Figure 3:**
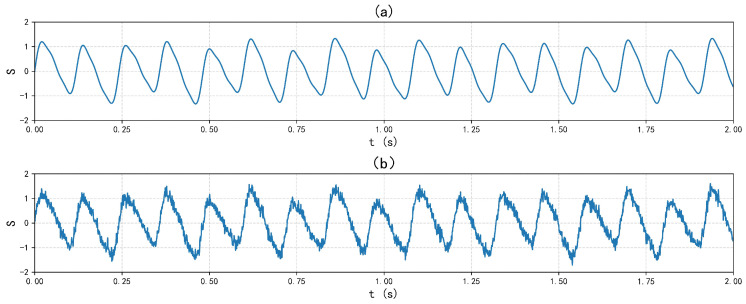
Time domain plot of the original simulated run-out signal.

Among them, the four main characteristic frequencies are $0.45f_{n}$, $1f_{n}$, $2f_{n}$, and $3f_{n}$ ($f_{n}$ is the unit’s rotational frequency), and $0.45f_{n}$ is the draft tube vortex band frequency. Assuming the rated speed of the pump turbine is 500r/min, the amplitudes of the four main characteristic frequencies are 5, 20, 5, and 2 respectively. Comparing these amplitudes with the maximum value of 20 simultaneously, the results are 0.25, 1, 0.25, and 0.1 respectively, serving as dimensionless amplitudes. n(t) is Gaussian white noise with a signal-to-noise ratio of 15dB. The original simulated run-out signal (a) and the noisy run-out signal (b) are constructed respectively, as shown in Figure 3 below. The signal sampling frequency is 1000Hz, and the sampling duration is 2s.

### 3.2. Signal Denoising Processing

**Figure 4:**
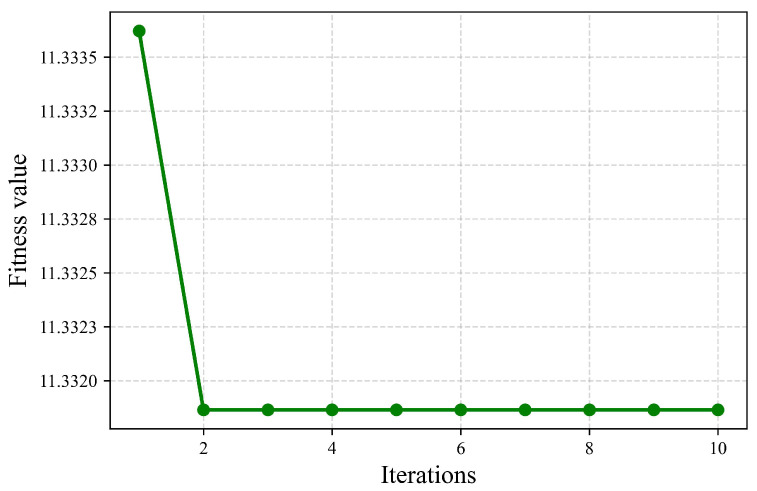
IPSO iterative optimization curve.

**Figure 5:**
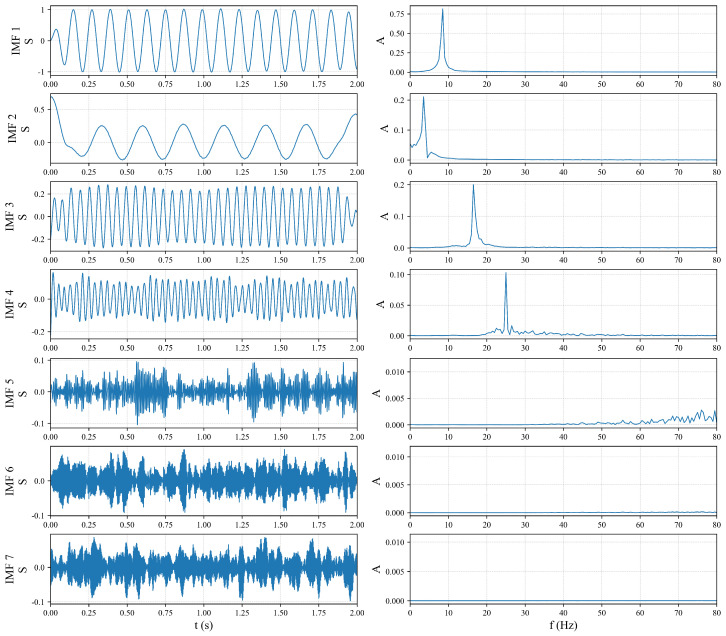
IPSO−VMD decomposition diagram.

According to the denoising process proposed above, first, the IPSO algorithm is used to optimize VMD parameters for the original signal. Among them, the value range of *K* is primarily determined based on the frequency composition characteristics of the run-out signal of the pumped-storage unit. The simulated run-out signal constructed in this paper includes the low-frequency component of the draft tube vortex band as well as main characteristic frequencies such as 1×, 2×, and 3× rotational frequencies. Therefore, the lower limit of *K* is set to 3 to ensure that the main vibration components can be effectively separated; meanwhile, to prevent an excessively large number of modes from causing over-decomposition, an increase in spurious modes, and a rise in computational complexity, the upper limit of *K* is set to 10. The penalty factor α is used to control the bandwidth of each modal component. If the value is too small, it is easy to cause the modal bandwidth to be too wide, resulting in mode mixing; if the value is too large, it may cause the mode to be too narrow, leading to excessive dispersion of effective frequency components. Comprehensively considering the distribution characteristics of the main rotational, harmonic, and hydraulic low-frequency components in the run-out signal of the pumped-storage unit, and combined with the results of multiple preliminary experiments, the search range for α is set to [100,5000] to balance decomposition accuracy, modal integrity, and computational efficiency. Regarding the parameter settings of the IPSO algorithm, while expanding the population size can enhance the global search capability of the algorithm, it significantly increases the computational overhead. Comprehensively considering the signal length processed in this paper and the computational efficiency, the population size is set to 50, which effectively controls the computational time while ensuring global search capability and stable algorithm convergence. In addition, through multiple convergence experimental tests, it is found that the algorithm can consistently reach convergence within 10 iterations. Therefore, setting the maximum number of iterations to 10 is strictly sufficient to ensure full convergence of the optimization process and guarantee the stability of the final optimized parameters, while effectively avoiding unnecessary computational redundancy. Due to the randomness in the iterative process, there is a certain error in the result of each optimization. After multiple iterative optimizations, the stable optimal parameter combination is obtained as [7,3221]. Its iterative optimization curve and VMD decomposition diagram are shown in Figure 4 and Figure 5 below, respectively.

**Figure 6:**
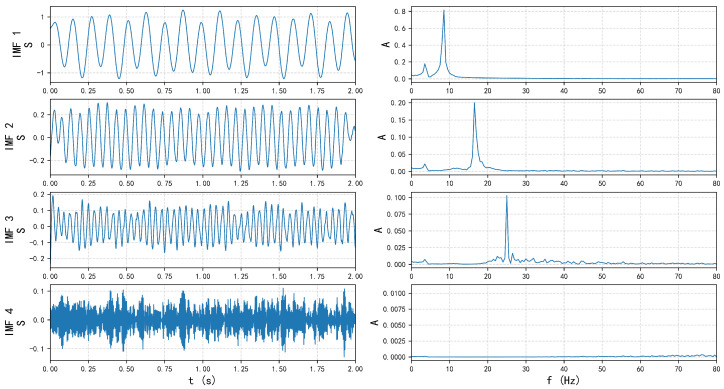
Default parameter VMD decomposition diagram.

Next, traditional VMD decomposition is performed on the original noisy signal. A set of default parameters [4,1500] is set, and the resulting decomposition diagram is shown in Figure 6 below.

From Figure 6, it can be seen that the VMD decomposition using default parameter settings did not effectively separate the main characteristic frequencies of the pump turbine signal in the obtained IMF components, and the phenomenon of mode mixing appeared. From the decomposition results after parameter optimization in Figure 5, it can be seen that the four main characteristic frequencies $0.45f_{n}$, $1f_{n}$, $2f_{n}$, and $3f_{n}$ are effectively separated out, facilitating subsequent analysis. This indicates that the IMF components optimized by the IPSO algorithm can more accurately express the original signal, with almost no mode mixing phenomenon.

According to the cross-correlation analysis method, the cross-correlation coefficients between each IMF component after VMD decomposition and the original noisy signal are calculated. The results are shown in Table 1. Combining Figure 5 and Table 1, it is known that after VMD decomposition, the adaptive partition of each IMF component is realized. Several main characteristic frequencies are decomposed into the first four IMF components, and according to Table 1, their correlation coefficients with the original signal are also relatively large, indicating that their degree of correlation is stronger, so they are classified as the effective mode group. The last three IMF components have a low correlation coefficient, and are classified as the noise modal component group.

According to the denoising process proposed in this paper, we comprehensively determined the correlation coefficient threshold by combining the cross-correlation coefficient table of each IMF component after VMD decomposition and the time–frequency distribution characteristic diagrams. As shown in Table 1, the preceding IMF components with higher cross-correlation coefficients with the original signal correspond to the effective components in Figure 5 that exhibit obvious periodic characteristics and match the rotational and harmonic frequency characteristics of the unit; while the components with cross-correlation coefficients below 0.1 show no obvious characteristic patterns in the time–frequency domain and are predominantly random noise. To eliminate the noise-dominated components while preserving the effective characteristic components, this paper selects a correlation coefficient threshold of 0.1, retaining only the IMF components with a correlation coefficient greater than 0.1 for subsequent secondary denoising processing. The noise-dominated modal components are directly removed. The correlation coefficient of IMF1 in the effective mode group is 0.9135, which has a very strong correlation with the original signal, so it is directly retained. The three effective components IMF2, IMF3, and IMF4 are subjected to wavelet threshold denoising. Secondary denoising is performed according to the wavelet denoising process mentioned above. The IMF1 and the denoised modal components are reconstructed to obtain the denoised signal. In this study, the selected wavelet basis function is db6, and the decomposition level is 6. The db6 wavelet possesses good orthogonality, compact support, and smoothness. It can effectively preserve the local transient information and periodic characteristics of the vibration signal while suppressing high-frequency noise, making it highly suitable for processing non-stationary vibration signals such as the run-out signals of pump turbines. The selection of the decomposition level is primarily determined based on the sampling frequency, the distribution of the main characteristic frequencies of the signal, and the feature preservation effect after denoising. Since the main effective frequencies in this paper are concentrated in the low-frequency region, employing six levels can achieve a reasonable division of the frequency band, avoiding both noise residue caused by too few decomposition levels and the loss of feature splitting caused by too many decomposition levels. Through comparative experimental verifications using four, five and seven levels, the denoising indicators corresponding to 6-level decomposition were found to be optimal. Therefore, this paper selects the db6 wavelet basis and 6-level decomposition to balance the smoothness of denoising and the capability to preserve local features.

### 3.3. Denoising Results Analysis

**Figure 7:**
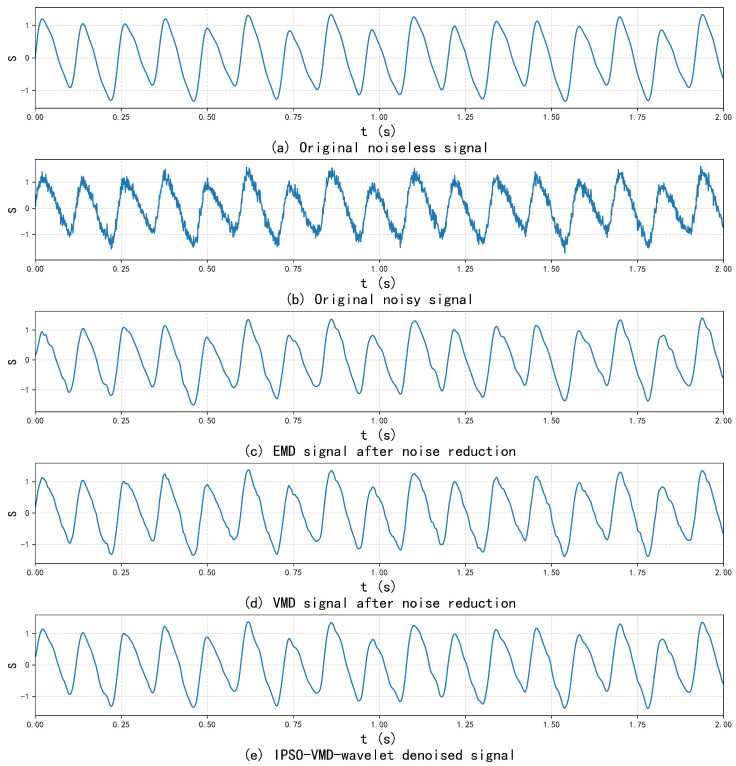
Denoising results of simulated run-out signal by different methods.

The default parameter VMD and the method proposed in this paper are used to denoise the simulated signal respectively, and the signal comparison results are shown in Figure 7 (the EMD denoising result is also given in the figure). The results show that the above methods can all effectively denoise the simulated noisy run-out signal. By observing the graph, it can be seen that the waveform curve obtained by the IPSO-VMD-Wavelet threshold denoising method proposed in this paper is smoother and closer to the original ideal signal waveform, achieving the expected denoising effect.

In order to further quantitatively evaluate the denoising effect of the method in this paper, signal-to-noise ratio (SNR) and root mean square error (RMSE) are introduced as evaluation indicators for this time.

SNR calculation formula:

(15) $$SNR=10\mathrm{lg}\left(\frac{\sum_{i=1}^{N}x_{i}^{2}}{\sum_{i=1}^{N}{\left(x_{i}-{\hat{x}}_{i}\right)}^{2}}\right)$$

RMSE calculation formula:

(16) $$RMSE=\sqrt{\frac{1}{N}\sum_{i=1}^{N}{\left(x_{i}-{\hat{x}}_{i}\right)}^{2}}$$

In the above formulas, $x_{i}$ is the original noisy signal; ${\hat{x}}_{i}$ is the denoised signal; *N* is the number of data points. The evaluation criteria are that the larger the SNR and the smaller the RMSE, the better the denoising effect.

According to the above calculation indicators, the calculation results for different denoising methods are shown in Table 2 (the table also gives the denoising results under the condition of a signal-to-noise ratio of 10 dB).

It can be seen from Table 2 that, compared with other methods, the IPSO-VMD-Wavelet threshold denoising method proposed in this paper obtains a higher signal SNR and a smaller RMSE under different SNR conditions, further illustrating the superiority of the denoising method proposed in this paper in the aspect of pump turbine simulated run-out signal denoising.

## 4. Measured Data Signal Denoising Analysis

**Figure 8:**
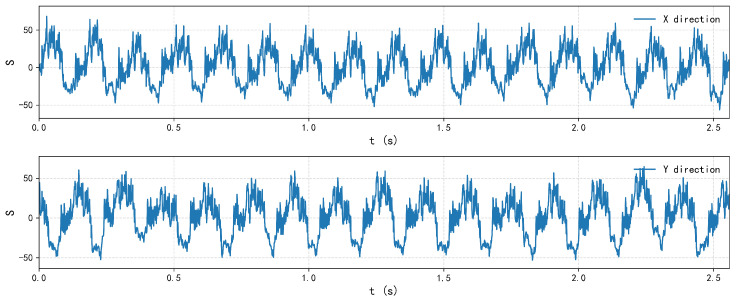
Original measured water guide run-out X and Y direction signal waveform.

This section uses the measured data collection of the run-out signal at the water guide bearing of a domestic pumped storage power station unit, including X and Y directions. The rated active power of the unit under generating conditions is 375 MW, and the rated rotational speed is 375 r/min, corresponding to a fundamental frequency of 6.25 Hz. The signal sampling frequency is set to 800 Hz. This paper intercepts a continuous sequence of 2048 data points for analysis. During this period, the real-time rotational speed of the unit is 100.3% of the rated speed, and the active power is −372 MW. Through calculation, the relative deviation of the rotational speed is only 0.3%, and the relative deviation of the load is 0.8%, with both rotational speed and load maintained near their rated values. Moreover, there are no load fluctuations or mode switching during this time period, indicating that the unit is in a typical steady-state pumping operation mode. Concurrently, the sampling duration corresponding to the 2048 sampling points is 2.56 s, with a frequency resolution of approximately 0.3906 Hz, which can clearly distinguish the fundamental frequency of the unit and its harmonic characteristics. This field-measured data accurately captures the typical vibration characteristics of pumped-storage units operating under stable pumping conditions, demonstrating strong representativeness. Its run-out signal time-domain waveform is shown in Figure 8.

**Figure 9:**
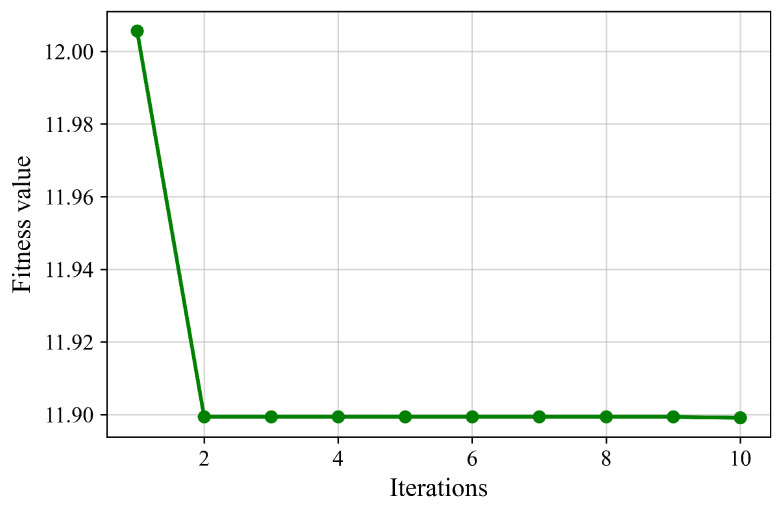
Water guide X-direction iterative optimization curve.

As can be seen from the figure, at this time, the unit has basically reached a steady state, but the signal is mixed with a large amount of noise. If the synthesis of the axis orbit is carried out directly, it will present a chaotic and irregular shape. Therefore, the denoising method proposed above is used to process the actual run-out signal. The optimal parameter combination obtained is [9,3398], and the iterative optimization curve is shown in Figure 9 below.

**Figure 10:**
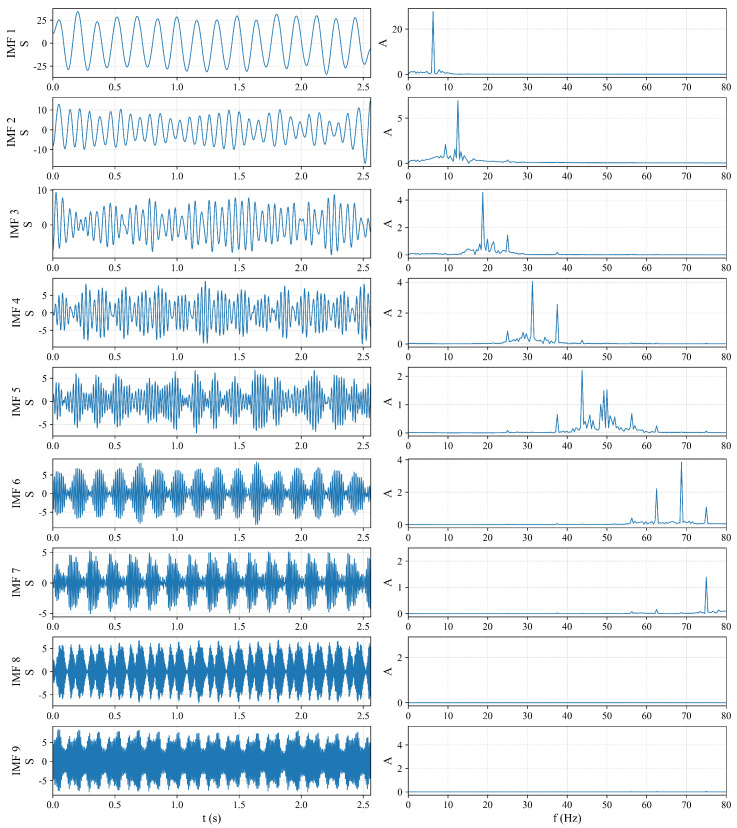
Measured water guide X−direction IPSO−VMD decomposition result.

The VMD decomposition is performed on the X-direction water guide run-out signal, and the result is shown in Figure 10. From Figure 10, it can be observed that after optimization decomposition, the phenomenon of mode mixing can be effectively avoided. Several important frequencies in the water guide bearing run-out signal, namely the 1× frequency, 2× frequency, and 3× frequency, are effectively separated into the first three IMF components.

**Figure 11:**
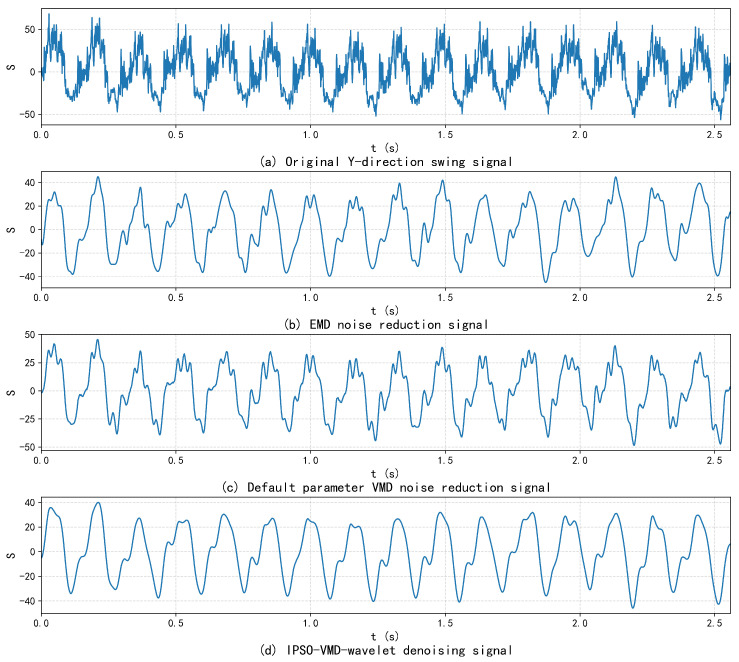
Comparison diagram of measured noise reduction results in X direction.

**Figure 12:**
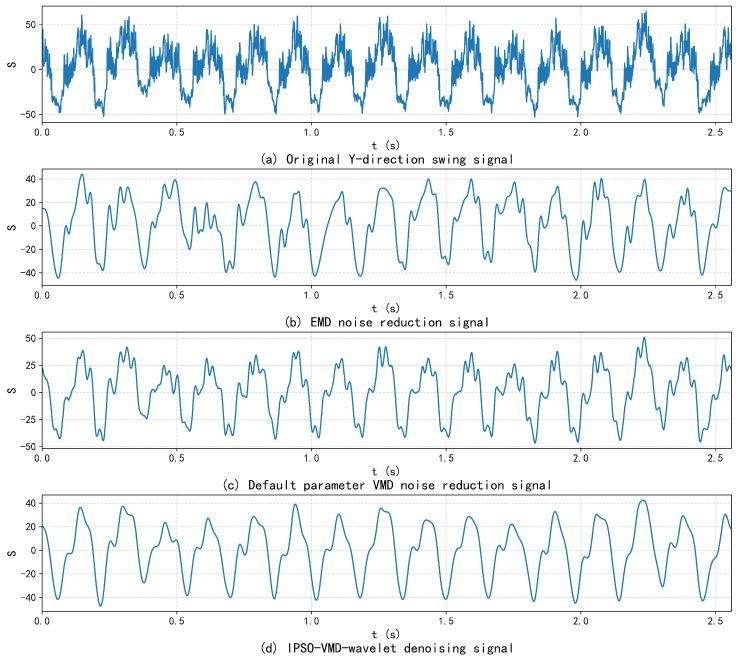
Comparison diagram of measured noise reduction results in Y direction.

Next, the cross-correlation coefficient analysis is performed on the decomposed IMF components, and wavelet threshold secondary denoising processing is carried out on them. The processing procedure is consistent with the above. Then, the effective modes are reconstructed to obtain the final denoised signal. At the same time, EMD and default parameter VMD decomposition are performed on the original measured water guide X-direction run-out signal for effect comparison. The results are shown in Figure 11. Similarly, EMD, VMD, and the method in this paper are used to denoise the measured run-out signal in the water guide Y-direction, and the results are shown in Figure 12.

It can be observed from Figure 11 and Figure 12 that the measured run-out signals have all achieved a certain denoising effect after being denoised by several methods. After EMD denoising, the waveform reflects the characteristics of the original measured signal on the whole, but there are some unsmooth bands. After default parameter VMD decomposition and denoising, there are more irregularities in the waveform. The waveform curve obtained after denoising by the IPSO-VMD-Wavelet denoising in this paper is smoother and more regular.

**Figure 13:**
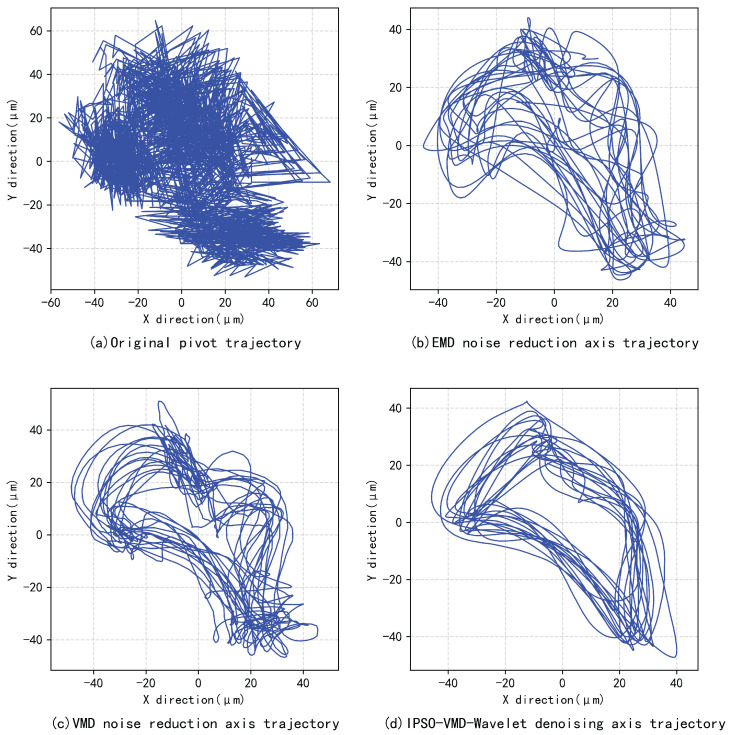
Axis orbit comparison diagram.

Figure 13 is the axis orbit obtained by synthesizing the X and Y directions of the water guide run-out. This figure shows the axis orbits before and after denoising based on the above methods. Under the steady-state operation of the unit, the regularity and periodic overlap rate of the axis orbit directly reflect the completeness of inherent vibration characteristics such as the rotational frequency. As can be seen from Figure 13, the original axis orbit contains large noise and has an irregular shape. However, the axis orbit curve obtained by denoising with the proposed method is smooth, has good closure, and exhibits a high periodic overlap rate without obvious distortion, proving that the periodic vibration characteristics, such as the 1× and 2× rotational frequencies, are not filtered out by noise and are all effectively preserved. Therefore, the denoising effect of this method is superior.

This paper also conducted a rigorous quantitative analysis in the frequency domain on the measured water guide bearing run-out signals in the X and Y directions before and after denoising. Local peak searching is performed within the local intervals of the target characteristic frequencies, and the frequency offsets and amplitude preservation rates before and after denoising in the X and Y directions of the water guide are calculated respectively as shown in Table 3 and Table 4. As illustrated in the tables, the offsets of several main characteristic frequencies of the pump turbine are 0Hz, and all amplitude preservation rates are greater than 99%. From a quantitative perspective, this clearly proves that the denoising method in this paper not only effectively filters out background noise but also retains useful characteristic frequencies with extremely high fidelity, and the positions of the characteristic peaks have no offset or distortion (no frequency leakage occurs).

Since the ideal noise-free signal cannot be obtained from the actual monitored signal of the power station, RMSE and SNR cannot be used as denoising evaluation indicators. Permutation entropy (PE) is introduced as the evaluation indicator for the measured run-out signal. PE is a calculation indicator for measuring the degree of data chaos. Its specific calculation formula is as follows:

(17) $$H_{P}\left(m\right)=-\sum P_{j}\ln P_{j}$$

where $P_{j}$ represents the probability distribution of the appearance of each permutation. Normalizing it yields the following formula:

(18) $$PE=\frac{H_{P}\left(m\right)}{\ln(m!)}$$

The larger the permutation entropy value, the more chaotic the signal; a smaller value indicates that the signal is more regular. When the entropy value is 0, it indicates that the signal is very regular and has no chaos. When it is 1, the signal is usually in a white noise state. In practical applications, the parameter embedding dimension *m* and time delay τ have a certain influence on the calculation result. In this paper, m=6 and τ=1.

The mean values of permutation entropy calculated for the denoised signals obtained using the above different denoising methods are shown in Table 5.

From the table, it can be seen that the mean value of permutation entropy for the water guide run-out signal denoising result based on the denoising method proposed in this paper is the smallest, achieving the optimal denoising effect.

In summary, compared with EMD and default parameter VMD, the denoising method proposed in this paper has obtained the optimal denoising effect, which facilitates deeper analysis of the signal in the later stage, and further proves the engineering practicality of the proposed method.

## 5. Summary and Outlook

Conclusions: To address the problem that monitoring signals from pumped-storage units are often heavily contaminated by intense background noise, making it difficult to effectively characterize the operational status of the equipment, this paper proposes a parameter-adaptive joint denoising method based on VMD and wavelet thresholding. First, parameter optimization using the IPSO algorithm is performed on the original signal to obtain the optimal decomposition parameters for VMD. Subsequently, cross-correlation analysis is conducted, followed by secondary denoising using wavelet thresholding. Finally, the processed components are reconstructed to obtain the final denoised signal. Both simulated run-out signals and actual monitoring signals from a domestic pumped-storage power station are utilized as experimental objects for signal denoising analysis. Through comparative analysis with other methods, the results demonstrate that the proposed method achieves optimal denoising performance, effectively eliminating background noise and significantly improving the signal-to-noise ratio (SNR). This study provides a reliable reference for the signal denoising processing and subsequent research and analysis of pumped-storage power stations, demonstrating significant engineering application value.

Outlook: Due to the limited space of this paper and the restriction of personal time and energy, the proposed signal denoising method has not yet been deeply applied in actual engineering scenarios. Therefore, combining the practical engineering development needs of the pumped-storage power station supporting the Yarlung Zangbo River Hydropower Station, the next step will focus on the extended application and optimal refinement of the pumped-storage units signal denoising method. The specific directions are as follows.

(1) Research on fault diagnosis of pumped storage units based on deep learning technology. Utilizing the denoising method proposed in this paper for the denoising preprocessing of sensor-acquired signals and the effective extraction of fault features, combined with deep learning models, an integrated diagnostic model of “denoising—feature extraction—fault recognition” will be constructed to enhance the precision and real-time performance of unit fault diagnosis.

(2) Fusion application of denoising methods and trend prediction of vibration or degradation of pumped storage units. Based on the proposed method, precise trend prediction models will be constructed using the denoised signals to realize accurate prediction of the pump turbine’s vibration state and equipment degradation process. This will enable advanced fault early warning for the equipment, provide scientific basis for the unit’s inspection and maintenance, and further enhance the operational reliability of pumped-storage units.

(3) Optimization research and application of denoising methods. Aiming at the special geological and hydrological environment of the Yarlung Zangbo River basin, as well as the complex conditions of the unit’s bi-directional operation, research will be conducted on the characteristics of the signals acquired by vibration sensors. This aims to further optimize the parameter optimization algorithm and enhance its adaptive capability under strong interference and complex operating conditions. Concurrently, exploration will be made into the integration of this denoising method with other signal processing technologies to further improve the signal denoising performance, thereby making it more tailored to practical engineering application scenarios.

## Figures and Tables

**Table 1:** Cross-correlation coefficients between each component and the original noisy signal.

IMF Component Index	Correlation Coefficient Value
1	0.9135
2	0.2994
3	0.2421
4	0.1193
5	0.0677
6	0.0708
7	0.0770

**Table 2:** Denoising indicators of different methods.

Denoising Method	SNR = 15 dB		SNR = 10 dB	
	RMSE	SNR/dB	RMSE	SNR/dB
Wavelet Denoising	0.1104	16.6697	0.1719	12.8231
EMD Denoising	0.0889	18.5475	0.1548	13.7324
VMD Denoising	0.0431	24.8294	0.0721	20.3744
Method in this Paper	0.0388	25.7577	0.0669	21.0201

**Table 3:** Quantitative evaluation of characteristic frequencies before and after denoising in the X direction.

Characteristic Frequency (Hz)	Original Peak Freq. (Hz)	Denoised Peak Freq. (Hz)	Frequency Offset (Hz)	Original Amp. (μm)	Denoised Amp. (μm)	Preservation Rate
1×	6.25	6.25	0	28.51	28.39	99.59%
2×	12.50	12.50	0	7.06	7.03	99.50%
3×	18.75	18.75	0	4.73	4.68	99.01%

**Table 4:** Quantitative evaluation of characteristic frequencies before and after denoising in the Y direction.

Characteristic Frequency (Hz)	Original Peak Freq. (Hz)	Denoised Peak Freq. (Hz)	Frequency Offset (Hz)	Original Amp. (μm)	Denoised Amp. (μm)	Preservation Rate
1×	6.25	6.25	0	29.76	29.71	99.85%
2×	12.50	12.50	0	7.15	7.12	99.68%
3×	18.75	18.75	0	6.19	6.17	99.57%

**Table 5:** Permutation entropy values of signals denoised by different methods.

Denoising Method	X Direction	Y Direction
EMD	0.2518	0.2630
VMD	0.3786	0.3686
IPSO-VMD-Wavelet Denoising	0.2179	0.2031

## Data Availability

The data presented in this study are available on request from the corresponding author.
